# Influence of time interval from diagnosis to treatment on survival for oral cavity cancer: A nationwide cohort study

**DOI:** 10.1371/journal.pone.0175148

**Published:** 2017-04-07

**Authors:** Wen-Chen Tsai, Pei-Tseng Kung, Yueh-Hsin Wang, Kuang-Hua Huang, Shih-An Liu

**Affiliations:** 1 Department of Health Services Administration, China Medical University, Taichung, Taiwan; 2 Department of Healthcare Administration, Asia University, Taichung, Taiwan; 3 Department of Otolaryngology, Taichung Veterans General Hospital, Taichung, Taiwan; 4 Faculty of Medicine, School of Medicine, National Yang-Ming University, Taipei, Taiwan; 5 Department of Medical Research, China Medical University Hospital, China Medical University, Taichung, Taiwan; Duke Cancer Institute, UNITED STATES

## Abstract

**Objectives:**

We aimed to explore the relationship between the time interval from diagnosis to treatment and survival of oral cavity squamous cell carcinoma patients.

**Materials and methods:**

A population-based study was conducted between 2004 and 2010. Claims data of oral squamous cell carcinoma patients were retrieved from the Taiwan Cancer Registry Database. Secondary data were obtained from Taiwan’s National Health Insurance Research Database.

**Results:**

A total of 21,263 patients were included in the final analysis. The majority of the patients received treatment within 30 days of diagnosis (n = 18,193, 85.5%), while 572 patients (2.7%) underwent treatment after 120 days. The patients who were treated after 120 days had a higher risk of death when compared to those treated within 30 days (Hazard ratio: 1.32, 95% Confidence intervals: 1.19 to 1.47).

**Conclusion:**

A longer time interval from diagnosis to treatment was found to be associated with a poorer prognosis among patients suffering from oral squamous cell carcinoma.

## Introduction

Squamous cell carcinoma is the most common histological type of oral cavity cancer. It is a growing global health issue, particularly in developing countries [1,2]. Oral cancer has a relatively low 5-year survival rate of 50% or less when compared with other major types of cancers, such as breast, skin, prostate, and urinary bladder cancers [3]. No significant breakthroughs have been made in the treatment of oral cavity squamous cell carcinoma (OCSCC) over the past decade. Although better combinations of loco-regional therapeutic protocols have improved patients’ quality of life, the 5-year overall survival rate of oral cavity cancer patients has not improved much in recent years [4].

Delay in cancer treatment can be classified into two main types, patient delay and treatment delay. Patient delay is defined as the duration from the patient’s first awareness of symptoms to the first visit to a physician, while treatment delay is the time interval between the date of confirmed diagnosis of cancer and the initiation of definitive treatment [3]. Lack of awareness, limited access to medical care, and long waiting lists due to manpower shortages were reported to be causes of treatment delay [1]. A previous study of breast cancer found that delays in both diagnosis and treatment were associated with a poor survival rate [5]. Another study also indicated that a prolonged time-to-treatment duration in diffuse large B-cell lymphoma was related to a lower progression-free survival rate [6]. Additionally, a recent large, population-based study found that longer waiting periods from diagnosis to treatment in uterine cancer patients had a negative influence on overall survival [7]. In terms of oral cavity cancer, delay in the start of treatment also influenced the survival rate according to an earlier case-control study [8]. A population-based retrospective study of head and neck cancer demonstrated that comorbid illnesses and professional delay were both independent prognosticators [9]. However, few studies have addressed the impact of treatment delay on the survival of oral cavity cancer patients in a population-based cohort. Therefore, the aim of this study was to investigate the association between time interval from diagnosis to initiation of treatment and survival of oral cavity cancer patients drawn from a nationwide database in Taiwan.

## Materials and methods

This study was approved by the Institutional Review Board of Cheng Ching Hospital Chung Kang branch (IRB number: HP150003) and was conducted in accordance with the Helsinki Declaration. All patients’ recognizable information was omitted prior to analysis. Taiwan is well-known worldwide for its comprehensive health care system, which is based on a national health insurance program which provides fair and equal access to its health services for all enrollees. There is a subset within the National Health Insurance Research Database (NHIRD) known as the Taiwan Cancer Registry Database (TCRD) which records all types of cancer diagnosed in Taiwan. We extracted the claims data of all patients diagnosed with oral cavity cancer from the TCRD. The NHIRD contains comprehensive health care information for 99.6% of Taiwan’s entire population of more than 23 million people [10]. The accuracy in the diagnosis of major diseases listed in the NHIRD, such as acute coronary syndrome and ischemic stroke, has been validated in previous studies [11,12].

### Selection of participants

We identified all patients who had been diagnosed with oral cavity cancer from the year 2004 to 2010 (International Classification of Diseases, Oncology, 3rd edition [ICD-O-3] codes: C00 –C06) as the parent group (excluding C01.9, C02.4, C05.1, and C05.2). We included patients with histology of SCC only (ICD-O-3, 805X ~ 808X). The follow-up end point was set at Dec. 31, 2012. The accuracy of diagnosis was validated by both ICD-O-3 codes and inclusion in the TCRD. Those who died within 1 month of their confirmed diagnosis, had distant metastases at diagnosis, had multiple primary cancers, carried incomplete data in the NHIRD and TCRD, or did not receive any treatment (such as surgery, chemotherapy or radiotherapy) were excluded.

### Description of variables

Demographic data, including gender and age at confirmation of diagnosis, was documented. Time interval from diagnosis to treatment was defined as the time from the date of diagnosis (date of biopsy which confirmed malignancy), until the date of initiation of the patient’s first treatment (surgery, radiotherapy, or chemotherapy). Tumor staging was categorized in accordance with the guidelines of the American Joint Committee on Cancer (6th edition for tumors diagnosed from 2004 through 2009, 7th edition for tumors diagnosed in 2010). Catastrophic illness/injury are listed in S1 Table. The urbanization levels ranged from highly developed urban cities (level 1) to remote districts (level 7). The number of services provided by primary hospitals was divided into low, medium and high categories based on the quartile (low: lowest quartile, medium: second and third quartile, high: highest quartile). Hospital ownership was classified as either public or private sector. The degree of comorbidity was categorized into three levels according to the Charlson Comorbidity Index (CCI) as modified by Deyo [13]. Other variables included patient’s monthly salary, treatment modalities (surgery, radiotherapy, and chemotherapy), hospital levels (medical centers, regional hospitals, and others), and multidisciplinary team (MDT) management.

### Main outcome measurements

The primary outcome measurement was overall survival. Follow-up duration was defined as the duration from the date of diagnosis until the date of death or follow-up endpoint. Death was identified as withdrawal of a patient from the National Health Insurance program and was validated by linking the administrative data set with the Taiwan Death Registry.

### Statistical analysis

We used descriptive statistics for general data presentation. Comparisons of nominal or ordinal variables between patients who were alive or dead were analyzed using the chi-square test, whereas continuous variables were examined by the Student’s *t* test. In addition, estimation of survival time was calculated by the Kaplan-Meier method, stratified by various tumor stages, in order to investigate the influences of diagnosis-to-treatment time interval on the survival of OCSCC patients. Furthermore, a univariate Cox proportional hazards regression was applied to analyze the prognostic factors for survival. Finally, modified Cox proportional hazard models were used to analyze the hazard ratio of the death of patients with various treatment delay durations after adjusting for age, sex, and other variables. All statistical analyses were performed using SAS software, version 9.2 (SAS Institute Inc., Cary, NC). A *P* value < 0.05 was regarded as statistically significant and all tests were two-sided.

## Results

There were 24,335 newly diagnosed OCSCC patients from 2004 to 2010 in Taiwan. Carcinoma in situ was found in 1,864 patients (7.7%) whereas 469 patients (1.9%) had multiple primary cancers or distant metastasis. In addition, 362 patients (1.5%) who did not receive any treatment during the follow-up period and 397 patients (1.6%) who had incomplete records were excluded. Consequently, 21,263 patients (87.3%) were included in our final analysis. The average age at the time of diagnosis was 53.5 (+ 12.0) years and the average follow-up period was 44.0 (+ 29.2) months. The average time interval from diagnosis to treatment was 24.3 (+ 76.4) days. In terms of primary subsites, tongue cancer (ICD-O-3 C02) accounted for over one-third of all patients (n = 7,643, 35.9%) while 4,232 patients (19.9%) had lip, gum, or mouth floor cancers (ICD-O-3 C00, C03-C04). There were 9,388 patients with cancer of the buccal mucosa, palate, or other unspecific sites (ICD-O-3 C05-C06) (44.1%). The majority of patients received surgical intervention as their first treatment (n = 19,799, 93.1%), followed by radiotherapy (n = 1,005, 4.7%) and chemotherapy (n = 459, 2.2%). Most of the patients underwent treatment within 30 days of diagnosis (n = 18,193, 85.6%). There were 2,498 patients (11.8%) who had their treatment between 31 to 120 days after diagnosis, while 572 patients (2.7%) received their treatment 120 days after diagnosis. Most of the patients presented with stage IV disease (n = 8,397, 39.5%), whereas stage I, II, and III diseases accounted for 5,979 (28.1%), 4,288 (20.2%), and 2,599 (12.2%) patients, respectively. Other detailed descriptive data are presented in Table 1.

**Table 1:** Descriptive statistics of oral cavity cancer patients with different time intervals from diagnosis to treatment.

Variables	Total no. of patients (% in column)	No. of patients (%)			*P* value
		< = 30 days (n = 18,193)	31 ~ 120 days (n = 2,498)	> 120 days (n = 572)	
**Follow up period, mean (SD), month**		44.7 (29.0)	39.7 (29.8)	39.1 (29.5)	< 0.001
**Gender**					0.010
Female	1,758 (8.3%)	1,489 (84.7%)	202 (11.5%)	67 (3.8%)	
Male	19,505 (91.7%)	16,704 (85.6%)	2,296 (11.8%)	505 (2.6%)	
**Age**					0.005
= < 44 years	5,001 (23.5%)	4,304 (86.1%)	567 (11.3%)	131 (2.6%)	
45 ~ 54 years	7,376 (34.7%)	6,337 (85.9%)	842 (11.4%)	197 (2.7%)	
55 ~ 64 years	4,988 (23.5%)	4,296 (86.1%)	554 (11.1%)	138 (2.8%)	
> = 65 years	3,898 (18.3%)	3,257 (83.6%)	535 (13.7%)	106 (2.7%)	
**Primary subsites (ICD-O-3)**					< 0.001
C02	7,643 (36.0%)	6,633 (86.8%)	815 (10.7%)	195 (2.5%)	
C05~C06	9,388 (44.2%)	8,019 (85.4%)	1,118 (11.9%)	251 (2.7%)	
C00, C03~C04	4,232 (19.9%)	3,541 (83.7%)	565 (13.4%)	126 (2.9%)	
**Other catastrophic illness/injury**					0.801
No	20,565 (96.7%)	17,594 (85.6%)	2,415 (11.7%)	556 (2.7%)	
Yes	698 (3.3%)	599 (85.8%)	83 (11.9%)	16 (2.3%)	
**Stage**					<0.001
I	5,979 (28.1%)	5,351 (89.5%)	532 (8.9%)	96 (1.6%)	
II	4,288 (20.2%)	3,753 (87.5%)	436 (10.2%)	99 (2.3%)	
III	2,599 (12.2%)	2,214 (85.2%)	316 (12.2%)	69 (2.6%)	
IV	8,397 (39.5%)	6,875 (81.9%)	1,214 (14.5%)	308 (3.6%)	
**Premium-based monthly salary (NTD)**					0.161
Low-income	446 (2.1%)	383 (85.9%)	54 (12.1%)	9 (2.0%)	
< = 17,280	1,030 (4.8%)	882 (85.6%)	116 (11.3%)	32 (3.1%)	
17,281~22,800	11,286 (53.1%)	9,589 (85.0%)	1,377 (12.2%)	320 (2.8%)	
> = 22,801	8,501 (40.0%)	7,339 (86.3%)	951 (11.2%)	211 (2.5%)	
**Urbanization level**					0.951
Level 1	4,918 (23.1%)	4,203 (85.5%)	587 (11.9%)	128 (2.6%)	
Levels 2 & 3	10,035 (47.2%)	8,576 (85.5%)	1,178 (11.7%)	281 (2.8%)	
Levels 4 & 5	3,969 (18.7%)	3,399 (85.6%)	469 (11.8%)	101 (2.6%)	
Levels 6 & 7	2,341 (11.0%)	2,015 (86.1%)	264 (11.3%)	62 (2.6%)	
**First treatment**					<0.001
Surgery	19,799 (93.1%)	17,239 (87.1%)	2,099 (10.6%)	461 (2.3%)	
Radiotherapy	1005 (4.7%)	654 (65.1%)	275 (27.4%)	76 (7.5%)	
Chemotherapy	459 (2.2%)	300 (65.4%)	124 (27.0%)	35 (7.6%)	
**Joint MDT within 180 days**					<0.001
No	18,068 (85.0%)	15,400 (85.2%)	2,124 (11.8%)	544 (3.0%)	
Yes	3,195 (15.0%)	2,793 (87.4%)	374 (11.7%)	28 (0.9%)	
**Charlson Comorbidity Index**					0.500
< 3	18,664 (87.8%)	15,946 (85.4%)	2,214 (11.9%)	504 (2.7%)	
4–6	1,875 (8.8%)	1,612 (86.0%)	213 (11.4%)	50 (2.6%)	
> 7	724 (3.4%)	635 (87.7%)	71 (9.8%)	18 (2.5%)	
**Hospital level**					<0.001
Medical centers	16,336 (76.8%)	14,037 (85.9%)	1,896 (11.6%)	403 (2.5%)	
Regional hospitals	4,815 (22.6%)	4,065 (84.4%)	589 (12.2%)	161 (3.4%)	
Others	112 (0.5%)	91 (81.3%)	13 (11.6%)	8 (7.1%)	
**Hospital ownership**					< 0.001
Public	5,891 (27.7%)	4,877 (82.8%)	850 (14.4%)	164 (2.8%)	
Private	15,372 (72.3%)	13,316 (86.6%)	1,648 (10.7%)	408 (2.7%)	
**Hospital services volume**					< 0.001
Low	5,474 (25.7%)	4,665 (85.2%)	603 (11.0%)	206 (3.8%)	
Middle	10,450 (49.2%)	8,901 (85.2%)	1,286 (12.3%)	263 (2.5%)	
High	5,339 (25.1%)	4,627 (86.7%)	609 (11.4%)	103 (1.9%)	

Abbreviations: SD, standard deviation; ICD-O-3, International Classification of Diseases, Oncology, 3rd edition; NTD, New Taiwan Dollar; MDT, multidisciplinary team

When we stratified the patients according to the time interval from diagnosis to treatment, there were no significant differences among the three groups in their premium-based monthly salary, urbanization level of hospitals, and CCI. However, the average follow-up period was longer for those treated within 30 days. In addition, patients > = 65 years, or those in advanced stages tended to be treated later. Also, patients treated initially with radiotherapy or chemotherapy were more likely to have a longer mean time interval from diagnosis to treatment, when compared with that of those who were treated initially with surgery. Moreover, patients treated in private hospitals had a shorter average time interval from diagnosis to treatment, when compared with those treated in public hospitals. Finally, patients who received treatment in hospitals with a low/medium service volume had a longer mean time interval from diagnosis to treatment when compared with that of those treated in hospitals with a high service volume. Detailed data are shown in Table 1.

When we categorized the patients based on their survival status, there was no significant difference between the two groups in receiving MDT management within 180 days. However, patients treated within 30 days tended to have a higher survival rate when compared with those treated after 30 days. In addition, female patients, younger age, primary site at the tongue, earlier stage diseases, higher premium-based monthly salary, treated initially by surgery, lower CCI, and treated in public hospitals were more likely to survive. Detail data are presented in Table 2.

**Table 2:** Descriptive statistics of oral cavity cancer patients based on survival status.

Variables	Total no. of patients (% in column)	No. of patients (%)		*P* value
		Survival group (n = 12,417)	Deceased group (n = 8,846)	
**Interval from diagnosis to treatment**				<0.001
= < 30 days	18,193 (85.5%)	10,994 (60.4%)	7,199 (39.6%)	
31 ~ 120 days	2,498 (11.8%)	1,218 (48.8%)	1,280 (51.2%)	
> 120 days	572 (2.7%)	205 (35.8%)	367 (64.2%)	
**Gender**				0.001
Female	1,758 (8.3%)	1,101 (62.6%)	657 (37.4%)	
Male	19,505 (91.7%)	11,316 (58.0%)	8,189 (42.0%)	
**Age**				<0.001
= < 44 years	5,001 (23.5%)	3,070 (61.4%)	1,931 (38.6%)	
45 ~ 54 years	7,376 (34.7%)	4,459 (60.5%)	2,917 (39.5%)	
55 ~ 64 years	4,988 (23.5%)	3,008 (60.3%)	1,980 (39.7%)	
> = 65 years	3,898 (18.3%)	1,880 (48.2%)	2,018 (51.8%)	
**Primary subsites (ICD-O-3)**				<0.001
C02	7,643 (35.9%)	4,635 (60.6%)	3,088 (39.4%)	
C05~C06	9,388 (44.2%)	5,381 (57.3%)	4,007 (42.7%)	
C00, C03~C04	4,232 (19.9%)	2,401 (56.7%)	1,831 (43.3%)	
**Other catastrophic illness/injury**				<0.001
No	20,565 (96.7%)	12,128 (59.0%)	8,437 (41.0%)	
Yes	698 (3.3%)	289 (41.4%)	409 (58.6%)	
**Stage**				<0.001
I	5,979 (28.1%)	4,819 (80.6%)	1,160 (19.4%)	
II	4,288 (20.2%)	2,972 (69.3%)	1,316 (30.7%)	
III	2,599 (12.2%)	1,470 (56.6%)	1,129 (43.4%)	
IV	8,397 (39.5%)	3,156 (37.6%)	5,241 (62.4%)	
**Premium-based monthly salary (NTD)**				<0.001
Low-income	446 (2.1%)	198 (44.4%)	248 (55.6%)	
< = 17,280	1,030 (4.8%)	602 (58.5%)	428 (41.5%)	
17,281~22,800	11,286 (53.1%)	6,357 (56.3%)	4,929 (43.7%)	
> = 22,801	8,501 (40.0%)	5,260 (61.9%)	3,241 (38.1%)	
**Urbanization level**				<0.001
Level 1	4,918 (23.1%)	2,957 (60.1%)	1,961 (39.9%)	
Levels 2 & 3	10,035 (47.2%)	5,919 (58.9%)	4,120 (41.1%)	
Levels 4 & 5	3,969 (18.7%)	2,225 (56.1%)	1,744 (43.9%)	
Levels 6 & 7	2,341 (11.0%)	1,320 (56.4%)	1,021 (43.6%)	
**First treatment**				<0.001
Surgery	19,799 (93.1%)	12,111 (61.2%)	7,688 (38.8%)	
Radiotherapy	1,005 (4.7%)	202 (20.1%)	803 (79.9%)	
Chemotherapy	459 (2.2%)	104 (22.7%)	355 (77.3%)	
**Joint MDT within 180 days**				0.299
No	18,068 (85.0%)	10,512 (58.2%)	7,556 (41.8%)	
Yes	3,195 (15.0%)	1,905 (59.6%)	1,290 (40.4%)	
**Charlson Comorbidity Index**				<0.001
< 3	18,664 (87.8%)	11,365 (60.9%)	7,299 (39.1%)	
4–6	1,875 (8.8%)	823 (43.9%)	1,052 (56.1%)	
> 7	724 (3.4%)	229 (31.6%)	495 (68.4%)	
**Hospital level**				0.001
Medical centers	16,336 (76.8%)	9,568 (58.6%)	6,768 (41.4%)	
Regional hospitals	4,815 (22.6%)	2,795 (58.1%)	2,020 (41.9%)	
Others	112 (0.5%)	54 (48.2%)	58 (51.8%)	
**Hospital ownership**				<0.001
Public	5,891 (27.7%)	3,541 (60.1%)	2,350 (39.9%)	
Private	15,372 (72.3%)	8,876 (57.7%)	6,496 (42.3%)	
**Hospital services volume**				<0.001
Low	5,474 (25.7%)	2,795 (51.1%)	2,679 (48.9%)	
Middle	10,450 (49.2%)	6,331 (60.6%)	4,119 (39.4%)	
High	5,339 (25.1%)	3,291 (61.6%)	2,048 (38.4%)	

Abbreviation: ICD-O-3, International Classification of Diseases, Oncology, 3rd edition; NTD, New Taiwan Dollar; MDT, multidisciplinary team

**Fig 1:**
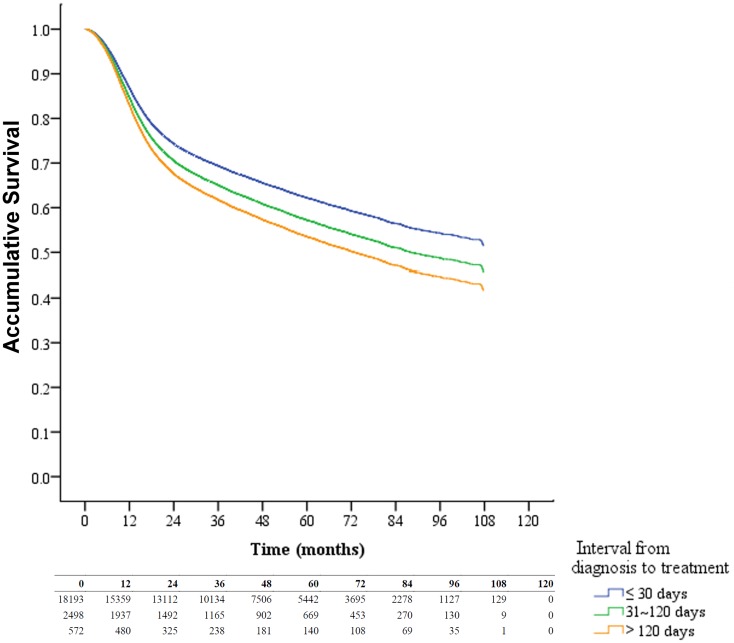
Overall survival curves of oral cavity squamous cell carcinoma patients stratified by different time intervals from diagnosis to treatment.

**Fig 2:**
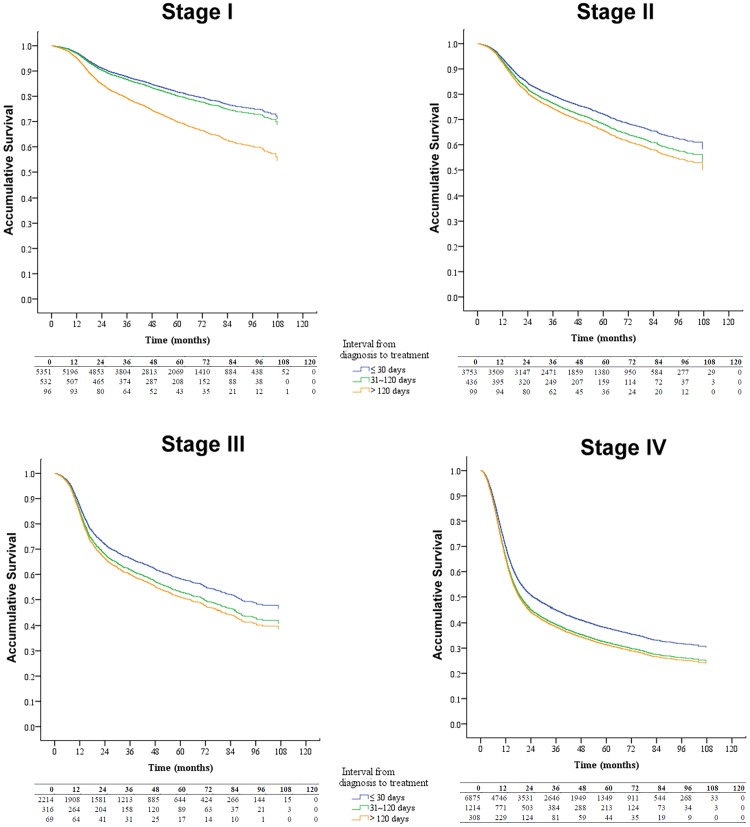
Overall survival curves of different time intervals from diagnosis to treatment based on tumor stage.

Using the Kaplan-Meier method, patients treated after 30 days from diagnosis had a lower overall survival rate when compared with those treated within 30 days (Fig 1). If we stratified the patients according to their initial tumor stage, the time interval from diagnosis to treatment remained a significant prognosticator (Fig 2). When compared with those treated within 30 days, patients treated after 120 days from diagnosis had a 1.32-fold increased risk of death (95% confidence interval: 1.19–1.47). Male gender, older age, other catastrophic illness/injury, late-stage diseases, low income, treated initially with radiotherapy/chemotherapy, higher CCI, or treated in private/low service volume hospitals were associated with a poorer prognosis. Detailed data are shown in Table 3.

**Table 3:** Factors associated with overall survival in oral cavity cancer patients.

Variables	No. of patients (N = 21,263)	Univariate HR (95% CI)	Multivariate HR (95% CI)
**Diagnosis to treatment time interval**			
< = 30 days	18,193	1.00 (ref)	1.00 (ref)
31 ~ 120 days	2,498	1.43 (1.35–1.52)	1.18 (1.11–1.25)
> 120 days	572	1.77 (1.59–1.96)	1.32 (1.19–1.47)
**Gender**			
Female	1,225	1.00 (ref)	1.00 (ref)
Male	15,466	1.15 (1.06–1.25)	1.20 (1.10–1.30)
**Age**			
= < 44 years	5,001	1.00 (ref)	1.00 (ref)
45 ~ 54 years	7,376	1.04 (0.98–1.10)	1.01 (0.96–1.07)
55 ~ 64 years	4,988	1.06 (1.00–1.13)	1.07 (1.00–1.14)
> = 65 years	3,898	1.54 (1.44–1.64)	1.55 (1.45–1.66)
**Primary subsites (ICD-O-3)**			
C02	3,756	1.00 (ref)	1.00 (ref)
C05~C06	2,874	1.11 (1.06–1.16)	0.87 (0.83–0.92)
C00, C03~C04	6,460	1.11 (1.04–1.17)	0.74 (0.69–0.78)
**Other catastrophic illness/injury**			
No	20,565	1.00 (ref)	1.00 (ref)
Yes	698	1.76 (1.60–1.95)	1.45 (1.31–1.61)
**Stage**			
I	5,979	1.00 (ref)	1.00 (ref)
II	4,288	1.66 (1.54–1.80)	1.62 (1.49–1.75)
III	2,599	2.65 (2.44–2.88)	2.55 (2.34–2.76)
IV	8,397	4.89 (4.59–5.22)	4.63 (4.34–4.95)
**Premium-based monthly salary (NTD)**			
ow-income	446	1.00 (ref)	1.00 (ref)
< = 17,280	1,030	0.62 (0.52–0.72)	0.80 (0.68–0.94)
17,281~22,800	11,286	0.67 (0.59–0.76)	0.83 (0.73–0.94)
> = 22,801	8,501	0.58 (0.51–0.66)	0.79 (0.69–0.90)
**Urbanization level**			
Level 1	4,918	1.00 (ref)	1.00 (ref)
Levels 2 & 3	10,035	1.04 (0.98–1.10)	1.02 (0.97–1.08)
Levels 4 & 5	3,969	1.14 (1.07–1.22)	1.01 (0.95–1.08)
Levels 6 & 7	2,341	1.14 (1.06–1.23)	1.06 (0.98–1.15)
**First treatment**			
Surgery	19,799	1.00 (ref)	1.00 (ref)
Radiotherapy	1,005	3.43 (3.19–3.69)	2.03 (1.88–2.19)
Chemotherapy	459	3.01 (2.71–3.35)	1.81 (1.63–2.02)
**Joint MDT within 180 days**			
No	18068	1.00 (ref)	1.00 (ref)
Yes	3195	0.97 (0.91–1.03)	0.93 (0.87–0.98)
**Charlson Comorbidity Index**			
< 3	18,664	1.00 (ref)	1.00 (ref)
4–6	1,875	1.66 (1.56–1.77)	1.38 (1.29–1.48)
> 7	724	2.38 (2.17–2.60)	1.71 (1.56–1.87)
**Hospital level**			
Regional hospitals	4,815	1.00 (ref)	1.00 (ref)
Medical centers	16,336	1.08 (1.02–1.13)	0.91 (0.86–0.97)
Others	112	1.38 (1.07–1.79)	1.03 (0.79–1.34)
**Hospital ownership**			
Public	5,891	1.00 (ref)	1.00 (ref)
Private	15,372	1.10 (1.05–1.15)	1.06 (1.01–1.11)
**Hospital services volume**			
Low	5,474	1.00 (ref)	1.00 (ref)
Middle	10,450	0.77 (0.73–0.81)	0.75 (0.71–0.80)
High	5,339	0.81 (0.77–0.86)	0.79 (0.74–0.85)

Abbreviations: ICD-O-3, International Classification of Diseases, Oncology, 3rd edition; NTD, New Taiwan Dollar; MDT, multidisciplinary team; HR, hazard ratio; CI, confidence interval

## Discussion

The influences of treatment delay on the prognosis of cancer patients varied among various primary sites. It was reported that a longer time interval between diagnosis and treatment was associated with shorter survival in breast cancer [5], uterine cancer, rectal cancer, bladder cancer, melanoma [7], and lung cancer patients [14]. However, a longer wait time was not related to the prognosis of esophageal cancer, gastric cancer, pancreatic cancer, colon cancer, renal cancer, or cervical cancer [7,15]. According to our population-based study, the time interval from diagnosis to treatment was an independent prognostic factor in OCSCC patients. A case-control study by van Harten et al. on head and neck squamous cell carcinoma (HNSCC) patients being treated in a Dutch comprehensive cancer center, found that treatment delay of up to 90 days was not associated with impaired survival [16]. However, their later study on HNSCC patients from the Netherland Cancer Registry Database revealed that a longer waiting time was significantly correlated with a higher mortality rate [17]. Another population-based study on HNSCC patients from the United States also indicated that time to treatment initiation of 61 to 90 days had a higher risk of death when compared with that of those with time to treatment initiation within 30 days [18]. A longer waiting time can possibly lead to progression of a tumor which makes treatment more difficult. A previous study found that the median duration of clinical upstaging from early stage to late stage was 11.3 months, whereas the average period from advanced tumor to untreatable tumor was 3.8 months [8]. This could explain why in the current study we found that patients with a time interval from diagnosis to treatment of more than 30 days had a 1.18 to 1.32-fold increased risk of death, when compared to those treated within 30 days.

Currently, the terms “treatment delay”, “wait time”, and “time interval from diagnosis to treatment” are used interchangeably. In our view, the application of the terms “treatment delay” and “wait time”, which are widely used in the literature, is not always appropriate as a portion of the time period is unavoidable [19]. Thus, we suggest that the phrase “time interval from diagnosis to treatment” is a more suitable term. An increased time interval from diagnosis to treatment can be partly blamed on the pursuit of improved health care [18]. Other possible explanations include advancements in pretreatment work-up [14], referrals to high service volume institutes [18], shortages of therapeutic instruments and manpower [1,15], pending second opinions requested by the patients or doctor [15], and a lack of education and public awareness [1,20]. All told, the reasons for the increased time interval from diagnosis to treatment of OCSCC patients remain multifaceted, integrated, and poorly understood [21].

In terms of gender, male patients tended to be treated earlier throughout our study, a finding which was not consistent with the results of previous studies [17,18]. Possible explanations include the different studied populations as well as the various social and cultural environments across the studies. The abovementioned studies included head and neck cancer patients, whereas our study included only oral cavity cancer patients. In addition, aesthetics is a considerable problem for patients after treatment of oral cavity cancer. It remains possible that female patients are more likely to be hesitant about receiving treatment after a detailed explanation is given to them regarding possible disfiguration. Interestingly, although female patients tended to be treated later, the prognosis for females was better than that for males in the current study. However, these results could have been confounded due to the dissimilar patient/disease features and different therapeutic protocols [10].

Although patients with tongue cancer were more likely to be treated within 30 days, the prognosis for tongue cancer patients was the poorest when compared with that for patients with primary cancer at other subsites. A previous study on oral cancer revealed that the prognostic factors and the failure patterns varied across different primary subsites [22]. Patients in more advanced stages tended to be treated later in this study. The results were comparable with previous population-based studies in both Denmark and the United States [17,18]. The abovementioned studies revealed that patients with a lower socio-economic status experienced a longer median waiting time when compared with those with a higher socio-economic status. However, our study found no relationship between time interval from diagnosis to treatment and premium-based monthly salary. This could be explained by the different health insurance systems. In Taiwan, patients with a confirmed diagnosis of cancer are officially certified as having a catastrophic illness and this certification exempts these individuals from any co-payment for cancer treatment. In addition, patients with low income are provided with subsidies, such as a waiver of deductibles, to guarantee their right to access proper health care [23].

Patients who undergo surgery as their first line of treatment tended to be treated within 30 days when compared with patients receiving radiotherapy/chemotherapy as their first treatment. Previous studies also found similar results [16–18]. Patients treated in medical centers, private hospitals, and high service volume hospitals had shorter time intervals from diagnosis to treatment. However, patients treated in public hospitals had a better survival when compared with those treated in private hospitals. Our previous study also reported similar results [10]. A previous study on the relationship between hospital volume/treatment delay and survival after cancer surgery indicated that improved outcomes were found in patients treated in high volume hospitals [24]. It was reported that many quality-of-care factors may contribute to a better prognosis in high service volume hospitals, including completeness of surgical resection, number of lymph nodes yielded, application of multimodality therapeutic protocol, and better treatment of comorbidities [24].

The Danish government implemented a fast track program in 2007 to minimize the time required for the diagnosis, staging and treatment of cancer patients [17]. In addition, the Dutch Head and Neck Society established a guideline which stated that 80% of head and neck cancer patients should be treated within 30 days after diagnosis [17]. The Brazilian Authority enacted a law in 2012 which specifies a maximum period of 60 days from diagnosis to treatment for cancer patients [6]. In the United States, a patient navigation model for head and neck cancer patients has been proposed to decrease the interval period between presentation to a clinic and treatment [25]. Since April 2013, Taiwan’s Ministry of Health and Welfare has subsidized hospitals which have implemented ‘‘MDT management for cancer patients” in order to improve the quality of cancer diagnosis and management [10]. In the current study, patients receiving MDT management within 180 days tended to be treated within 30 days. In addition, patients receiving MDT management within 180 days also had a better survival rate when compared with those who did not. MDT management facilitates the optimization of therapeutic protocols through the cooperation of surgeons, radiation oncologists, and medical oncologists [10].

Human papillomavirus (HPV) is closely associated with oropharyngeal cancer and a better prognosis is anticipated in HPV-related oropharyngeal cancer patients [26]. A recent meta-analysis indicated that the incidence of HPV-related oropharyngeal cancer patients was highest in Whites (61.1%), followed by 58.0% in Blacks and 25.2% in Asians [27]. Lee at al. in their prospective study indicated that HPV infections in advanced OCSCC patients are not uncommon and are clinically relevant. Interestingly, they found those with a single HPV-16 infection had a poor prognosis when compared with those with HPV-16-negative OSCC patients [28]. Nevertheless, the NHIRD/TCRD does not record HPV status and thus the relevant data could not be obtained for analysis in the current study.

A major strength of the current study is its relatively large sample size and resulting statistical power. In addition, our study population includes all OCSCC patients within Taiwan, and therefore any referral or selection biases that inevitably exist in traditional hospital-based observational studies would have been greatly minimized. However, there were certainly some limitations in the current study. First, it was not possible to definitively establish a causal relationship between time interval from diagnosis to treatment and survival of OCSCC patients in this observational study. Secondly, our databases (NHIRD and TCRD) do not include information on personal habits such as smoking status, alcohol abuse or frequency of betel quid chewing, as well as HPV status, all of which may have a negative impact on survival. Thirdly, our study only stratified patients based on their first treatment so the effects of combination treatments were not evaluated. Finally, information on recurrent diseases or causes of death was also not available in our database. Consequently, we were only able to calculate overall survival rather than disease-free survival or disease-specific survival.

In conclusion, although the causal relationship between time interval from diagnosis to treatment and survival requires further investigation, our results imply that shortening the time interval from diagnosis to treatment may improve the survival of OCSCC patients.

## Supporting information

S1 Table List of catastrophic illness/injury. (DOC)
