# Supplementary material for: Influence of time interval from diagnosis to treatment on survival for oral cavity cancer: A nationwide cohort study
Source: PLoS One. 2017 Apr 7;12(4):e0175148. doi: 10.1371/journal.pone.0175148 (PMC5384671; doi:10.1371/journal.pone.0175148)
Supplement: S1 Table — (DOC) [file pone.0175148.s001.doc]

**S1 Table. List of catastrophic illness/injury**

1. Malignant neoplasm
2. Congenital coagulation disorder
3. Hereditary/Acquired hemolytic anemias
4. Chronic renal failure
5. Systemic autoimmune diseases
6. Chronic psychotic conditions
7. Congenital metabolic disorders
8. Congenital anomalies or chromosomal anomalies
9. Burn of > 20% of total body surface or facial region
10. Organ replaced by transplant
11. Acute poliomyelitis with other paralysis or infantile cerebral palsy
12. Major trauma rated 16 or above on the severity scale
13. Long-term mechanical ventilation
14. Patients suffering from severe malnutrition due to major enterectomy, intestinal failure already on a fully intravenous diet for 30 days, and unable to obtain sufficient nutrition through an oral diet
15. Decompression sickness or air embolism
16. Myasthenia gravis
17. Congenital deficiency of immune system
18. Spinal cord injury
19. Occupational disease
20. Cerebrovascular disease
21. Multiple sclerosis
22. Congenital muscular dystrophy
23. Congenital anomalies integument
24. Leprosy (Hansen’s disease)
25. Liver cirrhosis with complication
26. Complications due to premature infants
27. Toxic effect of arsenic and its compounds (black foot disease)
28. Motor neuron disease
29. Jacob-Creutzfeldt disease
30. Rare disease
